# Pain Management and Parafunctional Activity Secondary to Local Anesthesia in Children 4–12 Years Old—A Retrospective Study

**DOI:** 10.3390/jcm14134623

**Published:** 2025-06-30

**Authors:** Aneta Olszewska, Agata Czajka-Jakubowska, Krzysztof Kujawa, Daniele Pergolini, Maurizio Bossù, Umberto Romeo, Jacek Matys

**Affiliations:** 1 Department of Orthodontics and Temporomandibular Disorders, Poznan University of Medical Sciences, 61-701 Poznan, Poland; ac-j@ump.edu.pl; 2 Statistical Analysis Center, Wroclaw Medical University, 50-367 Wroclaw, Poland; krzysztofkujawa@umw.edu.pl; 3 Department of Oral and Maxillofacial Sciences, University of Rome “Sapienza”, 00185 Rome, Italy; daniele.pergolini@uniroma1.it (D.P.); maurizio.bossu@uniroma1.it (M.B.); umberto.romeo@uniroma1.it (U.R.); 4 Department of Dental Surgery, Wroclaw Medical University, 50-425 Wroclaw, Poland; jacek.matys@umw.edu.pl; 5 Medical Center of Innovation, Wroclaw Medical University, Krakowska 26, 50-425 Wroclaw, Poland

**Keywords:** children, local anesthesia, parafunction, self-inflicted injury

## Abstract

**Objective**: Successful local anesthesia reduces patient pain and anxiety, facilitates the procedure, and enhances overall comfort; however, in children, it may also increase the risk of parafunctional activities in anesthetized areas. This retrospective study aimed to evaluate the factors influencing the risk of self-inflicted injuries. **Methods**: A study was conducted from January to December 2023 using the records of 4285 pediatric patients aged 4–12 years who underwent dental treatment under local anesthesia. Among 1161 cases at Poznan University (Poland), 73 (6.3%) of self-inflicted injuries were reported. At Rome University (Italy), 823 cases were reviewed, with 522 involving local anesthesia and 23 cases (4.4%) of trauma. **Results**: The overall prevalence of trauma following local anesthesia was 5.9%, indicating significant clinical concern. The lips were the most commonly affected (69.9%), followed by the cheeks (15.1%) and tongue (15.1%). The study confirmed a positive correlation between younger age and higher trauma incidence, with no significant differences in sex or ethnicity. Longer anesthesia duration and higher anesthetic doses were associated with increased lesion severity. The type of anesthetic also played a role, with articaine showing a higher risk compared to lidocaine. Furthermore, the type of injection influenced trauma incidence, due to the distribution of numbness and exploratory behaviors. **Conclusions**: Local anesthesia remains an indispensable tool in pain management in pediatric dentistry. However, the risk of self-inflicted injuries is not limited to the youngest patient. Proper education on post-anesthetic care is essential for minimizing complications and ensuring safe and effective dental treatment.

## 1. Introduction

Local anesthesia (LA) is defined as a temporary, reversible loss of sensation in a specific anatomical region, enabling pain-free and safe dental procedures in the stomatognathic area. The mechanism of action of local anesthetics involves the inhibition of nerve impulse transmission, achieved through the depression of neuronal excitability at nerve endings or the blockade of action potential propagation along peripheral nerves. This results in localized desensitization, preventing orofacial pain perception in the targeted area without impairing consciousness [1]. While local anesthesia is widely regarded as a safe and effective technique, it is not devoid of potential risks and adverse effects. Complications associated with dental local anesthesia can be categorized into immediate (or intraoperative) complications, occurring during administration in the dental office, and delayed (or postoperative) complications, which manifest after the patient has left the clinical setting [2]. Understanding these risks is essential for clinicians to optimize anesthetic techniques and enhance patient safety.

The resolution of local anesthesia is typically accompanied by mild discomfort at the injection site, which is generally of minimal clinical significance. However, in some cases, patients may experience persistent moderate to severe pain, which can last for several days [3]. Soft tissue anesthesia often extends beyond the duration of the dental procedure, increasing the risk of self-inflicted trauma, particularly in young children and individuals with developmental disabilities, who may inadvertently bite their lips, buccal mucosa (cheeks), or tongue due to altered sensation. When tissues are anesthetized, with the unfamiliar sensation of being numb, biting and chewing of lips and tongue as a self-inflicted injury can occur, especially in young children or ones who were unable to understand the procedure. Pain can be a protective phenomenon by which we tend to stay away from harmful activity. However, children can misinterpret altered sensation after local anesthesia due to a lack of normal proprioceptive feedback, often feeling restless and uncomfortable as a result, unconsciously continuing self-harming activity. It should be differentiated from iatrogenic secondary to local anesthesia lesions observed in the anesthetized area.

The prevalence of self-inflicted soft tissue injuries following local anesthesia has been estimated at 5% to slightly over 7%, depending on the study criteria [4]. However, other clinical studies reported a frequency of 13%, including not only severe cases but also small lesions such as redness or swelling [4,5]. The most commonly affected areas by biting were the lips (13%), buccal mucosa (10%), cheeks, and tongue (6%) [5].

Research has demonstrated that the frequency of self-inflicted injuries following local anesthesia is influenced by multiple factors, including patient age, duration of soft tissue anesthesia, and the anesthetic delivery method [6]. Younger children are particularly susceptible to self-inflicted trauma due to their limited cognitive ability, reduced cooperation, and lack of understanding of anesthesia-induced sensory changes [4]. Similar susceptibility has been observed in patients with intellectual disabilities, who may struggle with interpreting altered oral sensations, thereby increasing the risk of inadvertent biting injuries. The duration of soft tissue anesthesia is another critical factor. Prolonged anesthesia and numbness have been correlated with an increased likelihood of self-inflicted trauma, as patients may engage in repetitive biting or manipulation of the anesthetized tissue without experiencing immediate pain feedback [5,7]. Interestingly, the bilateral mandibular nerve block has been associated with a lower incidence of self-inflicted injuries compared to unilateral mandibular blocks. This phenomenon can be explained by two key factors: the reduced duration of soft tissue anesthesia upon patient discharge and the symmetrical distribution of numbness [4,8]. Bilateral mandibular blocks are typically administered in longer and more extensive treatments, leading to shorter residual anesthesia duration by the time the child leaves the dental office. Additionally, the symmetrical paresthesia on both sides of the oral cavity reduces the likelihood of exploratory behaviors, which are more commonly observed when unilateral numbness is present [4,9].

Late-onset complications following local anesthesia typically manifest after the anesthetic effect subsides and the patient has left the dental office. Among the most frequently observed complications are self-inflicted soft tissue injuries, which result from inadvertent biting of desensitized oral structures. These traumatic lesions often present as erythematous and inflamed areas, the severity of which depends on the location and extent of anesthesia. In mild cases, the injuries may cause transient discomfort, whereas more severe cases can lead to ulceration and significant pain. However, these lesions are generally self-limiting and heal spontaneously within two weeks, without long-term sequelae [10,11].

There are studies in the literature that assess injuries related to local anesthesia; however, these studies typically focus on a single population. In contrast, our work provides a comparative analysis of results from two different centers, which vary in socioeconomic, cultural, and demographic conditions.

Understanding the factors influencing self-inflicted soft tissue injuries following local anesthesia in pediatric patients is crucial for developing effective preventive strategies and optimizing clinical protocols to minimize adverse outcomes. The null hypothesis (H_0_) of the study states that patient age, anesthesia duration, anesthetic type, and injection method have no significant effect on the incidence or severity of self-inflicted trauma to the lips, cheeks, and tongue in children aged 4–12 years following local anesthesia.

## 2. Materials and Methods

The study was conducted in the Pediatric Dentistry Clinic, Poznan University of Medical Sciences (Poland) and the Department of Oral and Maxillofacial Sciences, University of Rome “Sapienza”, Rome (Italy) to assess the incidence of self-inflicted trauma secondary to local anesthesia in children.

A total of 4285 pediatric patients aged 4–12 years who had undergone dental treatment under local anesthesia were included. At Poznan University, 1161 cases of local anesthesia were identified, among which 73 cases (6.3%) involved self-inflicted injuries. At Sapienza University, 823 pediatric cases were reviewed, with 522 cases involving local anesthesia and 21 cases (4.02%) of trauma.

The study has been approved by the Bioethical Committee of the Poznan University of Medical Sciences (5 November 2022).

### 2.1. Subjects and Data Collection

During a 12-month period (January–December 2023), patient data were extracted from clinical charts from two universities: Pediatric Dentistry Clinic, Poznan University of Medical Sciences, and from the Department of Oral and Maxillofacial Sciences, University of Rome “Sapienza”, Italy. Inclusion criteria required that patients had undergone local anesthesia and had a follow-up visit scheduled within two days, where self-inflicted injuries could be recorded. The dental procedures performed included tooth extractions, conservative treatments, and pulp therapy.

The size of the study was determined by the observation period (12 months), the age of the patients, and the available information regarding traumatic injuries related to local anesthesia. A limitation of the study was that not all traumatic cases were recorded at the same clinic, as many patients traveled to the university clinic from other cities, which could affect the results. To assess the adequacy of the sample size, a post-hoc power analysis was performed for the observed age-related differences in trauma incidence (10% in children <6 years vs. 4% in those ≥6 years). The analysis indicated a medium effect size (Cohen’s h = 0.241) and a statistical power of 98.8%, confirming that the study was sufficiently powered to detect meaningful differences related to patient age.

### 2.2. Inclusion and Exclusion Criteria

Patients were eligible for inclusion if they met the following criteria:

- Age: 4–12 years old,
- Sex: both males and females,
- Cooperative behavior: patients scoring 3 or 4 on Frankl’s behavioral scale,
- Type of dental procedures: patients requiring local anesthesia for surgical, conservative, or pulp treatments,
- Dental history: patients attending follow-up visits after previous dental treatments (i.e., not their first dental visit),
- Cognitive and systemic health: children with normal intellectual development and no systemic or mental disorders,
- Follow-up adherence: patients who returned for follow-up visits at 2 days, 1 week, and 2 weeks post-treatment.

#### Exclusion Criteria

Patients were excluded if they met any of the following criteria:

- Parental refusal to sign informed consent for data inclusion,
- History of bleeding disorders or hypersensitivity to anesthetics,
- Use of analgesics within 12 h prior to the dental visit.

The evaluation of patients’ dental charts was conducted by two examiners, one at each center. The dental charts contained information gathered from interviews with parents, which included inquiries about the child’s behavior after leaving the office and their adherence to post-treatment recommendations (such as the duration of cheek numbness and when the child had their first meal after the dental appointment, etc.).

From a comprehensive clinical evaluation conducted and filled out in patient’s dental charts during follow-up visits, we gathered important information for analysis:

1. Medical Interview (Anamnesis):
  ○ General health status,
  ○ Previous history of self-inflicted injuries,
  ○ Prior dental experiences (positive or negative).
2. Extraoral Examination:
  ○ Assessment of lymph node changes, swelling, erythema, or skin alterations.
3. Intraoral Examination:
  ○ Evaluation of lesion size, number, and localization (severity),
  ○ Additional complaints, such as pain (measured using the Visual Analog Scale—VAS, if accessible), fever, or loss of appetite.

### 2.3. Statistical Analysis

All statistical analyses were performed using Statistica 13.3, licensed to Wrocław Medical University. Due to deviations from normal data distribution in most subclasses and small sample sizes in some groups, non-parametric tests were applied. The Mann–Whitney U test was used for comparisons between two independent variables, while Kruskal–Wallis test was applied for comparisons among more than two groups. Pearson’s chi-squared independence test with Yates’ continuity correction was utilized to assess relationships between categorical variables. Additionally, correlations between numerical variables were analyzed using Kendall’s Tau-b correlation coefficient, considering the presence of multiple tied ranks in the dataset. A *p*-value of <0.05 was considered statistically significant.

## 3. Results

### 3.1. Frequency of Self-Inflicted Injuries Following Local Anesthesia

The results of our study showed that self-inflicted injuries following local anesthesia occurred in 94 pediatric patients, with an average age of 6.2 years (range: 4–12 years). A total of 73 cases were recorded in Poznan and 21 cases in Rome, resulting in an overall prevalence of 5.9%. Although the incidence of traumatic lesions was higher in Poznan (6.7%) compared to Rome (4.2%), statistical analysis using the chi-square test (χ^2^ = 2.76, df = 1) showed that this difference was not statistically significant (*p* = 0.097) (Table 1).

### 3.2. Characteristics of Traumatic Injuries

The results of our study showed that the lips were the most commonly affected site (69.9%), followed by the cheeks (15.1%), and the tongue (15.1%). Statistical analysis revealed no significant association between lesion location and patient gender (*p* = 0.7406) (Table 2).

### 3.3. Association Between Injury Size and Anesthesia-Related Factors

**Figure 1:**
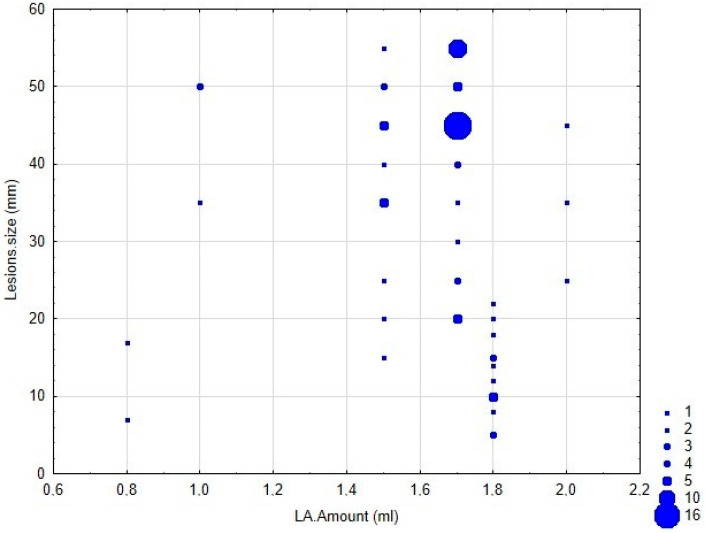
Correlation between lesion size and anesthetic amount (LA amount).

**Figure 2:**
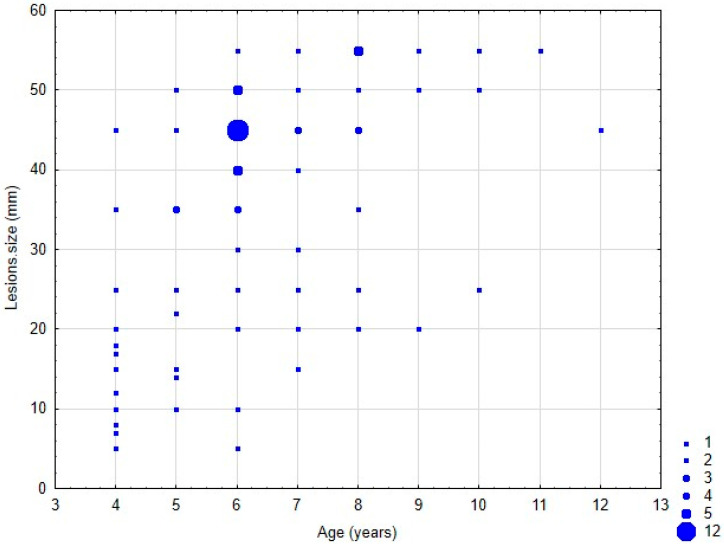
Correlation between lesion size (mm) and patients age (years).

The results of our study demonstrated that the size of self-inflicted injuries was significantly associated with all qualitative factors related to the anesthesia procedure, including anesthetic type, dosage, delivery type, and administration method (Table 3 and Table 4). However, the effects of ethnicity (*p* = 0.7809) and gender (*p* = 0.7456) on injury size were not statistically significant (Figure 1 and Figure 2).

### 3.4. Influence of Anesthesia Duration and Anesthetic Type on Lesion Location and Severity

The results of our study demonstrated that anesthetic type, longer anesthesia durations and higher anesthetic doses were associated with increased lesion location on the lips and their size (severity) (Figure 1). The type of anesthetic also influenced outcomes, with articaine showing a slightly higher association with injuries compared to lidocaine (Table 5). However, bupivacaine and mepivacaine were excluded from the analysis due to the small subclass size. Statistical analysis revealed that all observed relationships between lesion location and anesthetic type were not statistically significant (*p* = 0.7704).

### 3.5. Influence of Local Anesthesia Delivery Method on Self-Inflicted Injury Incidence and Severity

The results of our study demonstrated that the method of local anesthesia delivery influenced both the incidence and severity of self-inflicted injuries following dental treatment. A higher incidence of trauma was observed after infiltration anesthesia, which remains the most commonly used LA technique in children due to anatomical and clinical considerations. Interestingly, bilateral mandibular blocks were associated with a lower incidence of trauma compared to unilateral blocks (Table 6).

Furthermore, the comparison between conventional syringe, carpule injection, and the computer-controlled local anesthetic delivery system (CCLAD) revealed no statistically significant difference in the incidence of self-inflicted trauma among children aged 4–12 years (Table 7).

### 3.6. Correlation Between Age, Anesthetic Amount, and Lesion Frequency

The results of our study demonstrated a negative correlation between age (*p* = 0.2705), anesthetic amount (*p* = 0.4731), and lesion frequency, with younger patients exhibiting higher rates of self-inflicted trauma (Table 8).

Although no statistically significant differences were found between patient ethnicity and trauma incidence, clinical observations indicated that language barriers and limited communication skills contributed to a misunderstanding of post-procedural instructions, increasing the risk of injury, particularly among younger children who were unable to comprehend instructions independently (Table 9).

## 4. Discussion

As this study indicates, the anesthetized area in the oral cavity has a direct relation to pain and discomfort perception in children who cannot distinguish between pressure, pain, and other new sensations following local anesthesia. Oral mucosa and periodontal ligament have an abundant number of free nerve endings, whereas the submucosa area has fewer, making the injection of the anesthetic a source of many sensations arising in this particular tissue. If anatomical structure at different stages of child development and LA techniques are ignored, and if there is a wrong selection of anesthetic, there is an increased risk of adverse effects and complications, such as self-inflicted injury secondary to local anesthesia.

The prevalence of self-inflicted injuries following local anesthesia in children is significant, with studies reporting frequencies ranging from 4.4% to 13%, depending on the population and methodology [5].

The number of cases may often be underestimated because patients sometimes report to other specialists or clinics (pediatric, dermatologic, surgical, ER), where they are misdiagnosed with severe inflammation and may receive unnecessary treatments, including the use of antibiotics or general steroids [10].

Our study’s overall prevalence was 5.9%, which aligns with previous findings, estimating rates between 5% and 7% under similar conditions [12]. The lips were the most commonly affected site (69.9%), followed by the cheeks (15.1%) and the tongue (15.1%) [5]. These patterns are consistent with prior research, highlighting the lips’ vulnerability due to their prominent anatomical location and sensory alterations post-anesthesia [13]. Comparative analyses with studies by Adewumi et al. [5] and College et al. [12] demonstrate concordance regarding lesion localization but suggest variability in reported rates due to differences in study design and patient demographics. These findings underscore the importance of contextualizing prevalence data within specific clinical settings and populations.

Demographic factors play a critical role in the incidence of self-inflicted injuries. Younger children, particularly those under six, are more susceptible due to their limited understanding and communication abilities, as well as reduced awareness of the effects of anesthesia [14]. Our findings confirm a positive correlation between age and lesion frequency, with younger patients exhibiting higher trauma rates. Additionally, no significant differences were observed based on sex or ethnicity, suggesting that biological or cultural factors may exert less influence than developmental and behavioral characteristics [12].

Nevertheless, communication levels and understanding of post-procedural recommendations are crucial in preventing traumatic injuries secondary to local anesthesia. In the dental office, non-native parents/caregivers might misunderstand the guidance; sometimes older children are helpful in translation, but with younger ones, the issue is problematic. The dental office environment, which causes stress, anxiety, and tension in parents, often causes them to forget about the instructions given, not ask for their explanation, and want to leave the office as soon as possible [7]. Previous studies, such as those by College et al. [12], support these observations, highlighting the need for tailored approaches based on age and cognitive maturity rather than demographic categorizations.

The relationship between anesthetic variables and injury risk is complex. In our analysis, longer anesthesia durations and higher doses were associated with increased lesion severity [15]. The type of anesthetic also influenced outcomes, with articaine showing a slightly higher association with injuries compared to lidocaine, possibly due to its longer duration of action [16]. Interestingly, bilateral mandibular blocks exhibited a lower incidence of trauma than unilateral blocks, likely due to the symmetrical distribution of numbness reducing exploratory behaviors [5]. These findings align with research by Adewumi et al. [5], which emphasizes the role of both pharmacological and procedural factors in shaping post-anesthetic complications.

To minimize the risk of self-inflicted trauma, dentists should implement preventive clinical strategies and provide comprehensive post-procedural guidance to both children and their caregivers [17]. Effective communication should emphasize the expected sensory alterations following local anesthesia and educate patients on strategies to prevent accidental injury. Specific recommendations include avoiding mastication, refraining from consuming very hot beverages, and minimizing activities that could exert mechanical stress on anesthetized tissues while the numbing effect persists [13]. Symptoms such as tingling, numbness, dullness, or swelling of the lips should be recognized as indicators of residual anesthesia. In cases where patient cooperation is limited, the placement of a cotton roll or similar protective barrier can serve as a physical reminder to prevent inadvertent biting of the lips or cheeks [7]. However, the use of cotton rolls, recommended in the literature, as associated with the risk of aspiration or swallowing should be replaced by the other methods.

These measures are essential in ensuring a safe and complication-free recovery following local anesthesia in pediatric dental patients. In situations where the risk of self-inflicted soft tissue injury is particularly high, the use of phentolamine mesylate (OraVerse^®^) may be considered as a pharmacological intervention [15]. This vasodilatory agent, administered via injection at the same site as the anesthetic, accelerates the reversal of soft tissue anesthesia, thereby reducing the duration of numbness and minimizing the likelihood of injury [8,9]. However, its use in young pediatric patients presents challenges as it requires an additional injection, which may be poorly tolerated [15].

The double-blind, controlled trial conducted by Hersh et al. on phentolamine mesylate in young dental patients aged two to five years yielded promising results concerning the safety of the medication. While not achieving statistical significance, this study may have identified a potential safety signal related to systolic and diastolic blood pressure, as well as a possible occurrence of reflex tachycardia in this vulnerable patient population [18].

Furthermore, concerns have been raised regarding its potential systemic effects and toxicity, necessitating a careful risk-benefit assessment before its routine application in children [11]. Evidence-based guidelines emphasizing age-appropriate communication and monitoring are crucial in minimizing the risk of self-harm while enhancing overall patient safety [17,19].

This study has several limitations that warrant consideration. The retrospective design and reliance on clinical records may introduce biases, such as underreporting minor injuries or variations in documentation practices [12,20]. The sample size, while substantial, may not fully capture the diversity of pediatric populations, limiting the generalizability of our findings [14,21]. Furthermore, the study’s 12-month observation period restricts the ability to assess long-term outcomes or trends [15,22].

## 5. Conclusions

The results of our research indicate that the patient age, anesthesia duration, anesthetic type, and injection method have no significant effect on the incidence of self-inflicted trauma to the lips, cheeks, and tongue in children aged 4–12 years following local anesthesia.

However, the size of self-inflicted injuries was significantly associated with all qualitative factors related to the anesthesia procedure, including anesthetic type, dosage, delivery type, and administration method.

Future research should focus on prospective designs, larger and more diverse cohorts, and the development of standardized protocols for preventing and managing self-inflicted injuries in children following local anesthesia.

## Figures and Tables

**Table 1:** Study group of patients 4–12 years old scheduled for dental treatment in 2023 (from January to December) in two universities.

Patients	Poznan		Rome		Total	
	N	%	N	%	N	%
Without trauma	1088	93.3	501	95.8	1589	94.1
Self-inflicted injury	73	6.7	21	4.2	94	5.9
Total	1161	100.0	522	100.0	1683	100.0

Chi-square test: χ^2^ = 2.76, df = 1, *p* = 0.097.

**Table 2:** Characteristics of traumatic lesion location.

Lesion Location	Females		Males		Total	
	N	%	N	%	N	%
Lips	27	69.2	38	70.4	65	69.9
Cheeks	7	17.9	7	13.0	14	15.1
Tongue	5	12.8	9	16.7	14	15.1
Total	39	100.0	54	100.0	93	100.0

Chi-square test: χ^2^ = 0.60, df = 2, *p* = 0.7406.

**Table 3:** Effects of sex, ethnicity, anesthetic features, and city on the lesion size in the Mann–Whitney test.

Factor	Sample Size (N)		Median (Q1, Q3)		Z	*p*
	Females	Males	Females	Males		
Sex	40	54	37 (37, 45)	40 (40, 45)	−0.32	0.7456
Ethnicity	34	60	40 (40, 45)	40 (40, 50)	−0.28	0.7809
LA Anesthetic	29	60	15 (15, 25)	45 (45, 50)	−5.25	0.0000
Injection type	44	46	45 (45, 45)	27.5 (27.5, 45)	2.40	0.0163
LA delivery type	42	51	21 (21, 45)	45 (45, 50)	−3.41	0.0006
City	73	21	45 (45, 50)	12 (12, 15)	6.87	0.0000

Z—test value, *p*—statistical significance.

**Table 4:** Kendall’s Tau-b correlation between the lesion size and LA amount and age.

Correlated Variables	N	Tau-b	Z	*p*
Lesions.size and LA.Amount	93	−0.309	−4.39	0.00001
Lesions.size and Age	94	0.437	6.24	0.00000

N—sample size, Z—test value, *p*—statistical significance.

**Table 5:** Characteristics of lesion location and LA anesthetic type (L—lidocaine, A—articaine, B—bupivacaine, M—mepivacaine).

Lesion Location	L		A		B		M		Total	
	N	%	N	%	N	%	N	%	N	%
Lips	20	71.4	43	71.7	1	25.0	1	100.0	65	69.9
Cheeks	5	17.9	8	13.3	1	25.0	0	0.0	14	15.1
Tongue	3	10.7	9	15.0	2	50.0	0	0.0	14	15.1
Total	28	100.0	60	100.0	4	100.0	1	100.0	93	100.0

Chi-square test: χ^2^ = 0.52, df = 2, *p* = 0.7704.

**Table 6:** Contingency table for lesion location and injection type.

Lesion Location	Infiltration		Block		Intraosseous		Total	
	N	%	N	%	N	%	N	%
Lips	34	77.3	29	64.4	2	66.7	65	70.7
Cheeks	5	11.4	8	17.8	1	33.3	14	15.2
Tongue	5	11.4	8	17.8	0	0.0	13	14.1
Total	44	100.0	45	100.0	3	100.0	92	100.0

Chi-square test: χ^2^ = 1.770428, df = 2, *p* = 0.4127; Injection type “Intraosseus” was excluded from the analysis because of small subclass size.

**Table 7:** Contingency table for lesion location and LA delivery type.

Lesion Location	Carpule		CCLAD		Total	
	N	%	N	%	N	%
Lips	27	65.9	37	72.5	64	69.6
Cheeks	7	17.1	7	13.7	14	15.2
Tongue	7	17.1	7	13.7	14	15.2
Total	41	100.0	51	100.0	92	100.0

Chi-square test: χ^2^ = 0.48, df = 2, *p* = 0.7861.

**Table 8:** Relationships between age, the LA amount, and lesion location in the Kruskal–Wallis test.

Variable	Sample Size (N)				Median (Q1, Q3)		H	*p*
	Lips	Cheeks	Tongue	Lips	Cheeks	Tongue		
Age	65	14	14	6.0 (5.0, 8.0)	6.5 (5.0, 9.0)	6.0 (6.0, 6.0)	2.62	0.2705
LA.Amount	64	14	14	1.7 (1.5, 1.7)	1.7 (1.5, 1.8)	1.7 (1.5, 1.7)	1.50	0.4731

H—test value, *p*—statistical significance.

**Table 9:** Ethnicity (communication level): The ratio native/others in both centers were without statistical significance.

Ethnicity	Rome	Poznan	Total
native	7	27	34 (36.1%)
others	14	46	60 (63.9%)

Pearson’s chi-squared test with Yates’ continuity correction: X-squared = 0.0024347, df = 1, *p*-value = 0.9606, and Pearson’s chi-squared test (without correction): X-squared = 0.094261, df = 1, *p*-value = 0.7588.

## Data Availability

The original contributions presented in this study are included in the article. Further inquiries can be directed to the corresponding author(s).

## Abbreviations

The following abbreviations are used in this manuscript:

LA	local anesthesia
CCLAD	computer controlled local anesthesia delivery

## References

[1] John R.R., Bonanthaya K., Panneerselvam E., Manuel S., Kumar V.V., Rai A. (2021). Local anesthesia in oral and maxillofacial surgery. Oral and Maxillofacial Surgery for the Clinician.

[2] Lipovsek M., Quereshy F.A., Shah M. (2020). Local anesthesia in oral and maxillofacial surgery. Oral Maxillofac. Surg. Clin. N. Am..

[3] Chelly J.E., Ghisi D. (2017). Local anesthetic nerve injury: Incidence, causes, prevention, and management. Reg. Anesth Pain Med..

[4] College C., Feigal R., Wandera A., Strange M. (2000). Bilateral versus unilateral mandibular block anesthesia in a pediatric population. Pediatr. Dent..

[5] Adewumi A., Hall M., Guelmann M., Riley J. (2008). The incidence of adverse reactions following 4% septocaine (Articaine) in children. Pediatr. Dent..

[6] Parate K., Mohod S.C. (2022). Soft Tissue Injuries after Administration of Local Anaesthesia. J. Res. Med. Dent. Sci..

[7] Chicka M.C., Dembo J.B., Mathu-Muju K.R., Nash D.A., Bush H.M. (2012). Adverse events during pediatric dental anesthesia and sedation: A review of closed malpractice insurance claims. Pediatr. Dent..

[8] Hersh E.V., Moore P.A., Papas A.S., Goodson J.M., Navalta L.A., Rogy S., Rutherford B., Yagiela J.A. (2008). Soft Tissue Anesthesia Recovery Group. Reversal of soft-tissue local anesthesia with phentolamine mesylate in adolescents and adults. J. Am. Dent. Assoc..

[9] Tavares M., Goodson J.M., Studen-Pavlovich D., Yagiela J.A., Navalta L.A., Rogy S., Rutherford B., Gordon S., Papas A.S. (2008). Reversal of soft-tissue local anesthesia with phentolamine mesylate in pediatric patients. J. Am. Dent. Assoc..

[10] Chi D., Kanellis M., Himadi E., Asselin M.-E. (2008). Lip biting in pediatric dental patients following dental local anesthesia: A case report. J. Pediatr. Nurs..

[11] Rutherford B., Zeller J.R., Thake D. (2009). Local and systemic toxicity of intraoral submucosal injections of phentolamine mesylate (OraVerse). Anesth. Prog..

[12] College C.W., Jackson D., Smith R. (2012). Patterns and prevalence of post-anesthetic soft tissue injuries in children. J. Clin. Ped. Dent..

[13] Haas D.A. (2002). An update on local anesthetics in dentistry. J. Can. Dent. Assoc..

[14] Malamed S.F. (2010). Handbook of Local Anesthesia.

[15] Hersh E.V., Moore P.A., Papas A.S. (2008). Pharmacologic management of dental pain: The role of nonsteroidal anti-inflammatory drugs and opioids. Dent. Clin. N. A.

[16] American Academy of Pediatric Dentistry (2021). Guidelines on the use of local anesthesia for pediatric dental patients. Pediatr. Dent..

[17] Lipp M., Dick W., Daubländer M. (1993). Efficacy and safety of articaine versus lidocaine in dental local anesthesia: A meta-analysis. J. Clin. Anesth..

[18] Hersh E.V., Lindemeyer R., Berg J.H., Casamassimo P.S., Chin J., Marberger A., Lin B.P., Hutcheson M.C., Moore P.A. (2017). Phase Four, Randomized, Double-Blinded, Controlled Trial of Phentolamine Mesylate in Two- to Five-year-old Dental Patients. Pediatr. Dent..

[19] Kot K., Krawczuk-Molęda E., Marek E., Lipski M. (2018). Self-inflicted injury as a complication following dental local anaesthesia in children—Case reports. J. Stomatol..

[20] Ho J.-P.T.F., van Riet T.C.T., Afrian Y., Sem K.T.C.J., Spijker R., de Lange J., Lindeboom J.A. (2021). Adverse Effects Following Dental Local Anesthesia: A Literature Review. J. Dent. Anesth. Pain Med..

[21] Bagattoni S., D’Alessandro G., Gatto M.R., Piana G. (2020). Self-Induced Soft-Tissue Injuries Following Dental Anesthesia in Children with and without Intellectual Disability. A Prospective Study. Eur. Arch. Paediatr. Dent. Off. J. Eur. Acad. Paediatr. Dent..

[22] Alghamidi W.A., Alghamdi S.B., Assiri J.-A., Almathami A., Alkahtani Z., Togoo R. (2019). Efficacy of Self-Designed Intraoral Appliances in Prevention of Cheek, Lip and Tongue Bite after Local Anesthesia Administration in Pediatric Patients. J. Clin. Exp. Dent..

